# Differentiation between young adult Internet addicts, smokers, and healthy controls by the interaction between impulsivity and temporal lobe thickness

**DOI:** 10.1556/2006.8.2019.03

**Published:** 2019-02-11

**Authors:** András N. Zsidó, Gergely Darnai, Orsolya Inhóf, Gábor Perlaki, Gergely Orsi, Szilvia Anett Nagy, Beatrix Lábadi, Kata Lénárd, Norbert Kovács, Tamás Dóczi, József Janszky

**Affiliations:** 1 Institute of Psychology, University of Pécs, Pécs, Hungary; 2 Department of Neurology, Medical School, University of Pécs, Pécs, Hungary; 3 MTA-PTE Clinical Neuroscience MR Research Group, Pécs, Hungary; 4 Pécs Diagnostic Centre, Pécs, Hungary; 5 Department of Neurosurgery, Medical School, University of Pécs, Pécs, Hungary; 6 MTA-PTE Neurobiology of Stress Research Group, Szentágothai Research Center, Pécs, Hungary

**Keywords:** Barratt Impulsiveness Scale, BIS-11, cortical thickness, middle temporal cortex, problematic Internet use, smokers

## Abstract

**Background and aims:**

Internet addiction is a non-substance-related addiction disorder with progressively growing prevalence. Internet addiction, like substance-related addictions, has been linked with high impulsivity, low inhibitory control, and poor decision-making abilities. Cortical thickness measurements and trait impulsivity have been shown to have a distinct relationship in addicts compared to healthy controls. Thus, we test whether the cortical correlates of trait impulsivity are different in Internet addicts and healthy controls, using an impulsive control group (smokers).

**Methods:**

Thirty Internet addicts (15 females) and 60 age- and gender-matched controls (30 smokers, all young adults aged 19–28 years) were scanned using a 3T MRI scanner and completed the Barratt Impulsiveness Scale.

**Results:**

Internet addicts had a thinner left superior temporal cortex than controls. Impulsivity had a significant main effect on the left pars orbitalis and bilateral insula, regardless of group membership. We identified divergent relationships between trait impulsivity and thicknesses of the bilateral middle temporal, right superior temporal, left inferior temporal, and left transverse temporal cortices between Internet addicts and healthy controls. Further analysis with smokers revealed that the left middle temporal and left transverse temporal cortical thickness change might be exclusive to Internet addiction.

**Discussion:**

The effects of impulsivity, combined with a long-term exposure to some specific substance or stimuli, might result in different natures of relationships between impulsivity and brain structure when compared to healthy controls.

**Conclusion:**

These results may indicate that Internet addiction is similar to substance-related addictions, such that inefficient self-control could result in maladaptive behavior and inability to resist Internet use.

## Introduction

Internet addiction (IA; other proposed terms are “problematic Internet use,” “pathological Internet use,” or “excessive Internet use”) is a non-substance-related addiction disorder that is becoming increasingly common among adolescents and young adults, and has become an epidemic worldwide (Block, 2008). According to recently proposed diagnostic criteria, it is characterized by preoccupation, withdrawal, craving, loss of control, excessive use, loss of interests, and escape or relief from a dysphoric mood (Kuss & Lopez-Fernandez, 2016). The prevalence of IA before 2010 was estimated to range from approximately 1% to 14% (Aboujaoude, 2010; Block, 2008), while less than a decade later the prevalence of IA had risen drastically and was estimated to range from 4% to as much as 46% in some countries (Islam & Hossin, 2016; Mak et al., 2014). However, the true prevalence is hard to estimate because of high cross-cultural variance.

Studies have identified severe consequences of IA, such as sleep deprivation, academic under-achievement, decreased ability to concentrate, obesity, symptoms similar to those of attention-deficit hyperactivity disorder, self-injuring behavior, suicidal attempts, substance misuse, and short-term goal orientation (Aboujaoude, 2017; Durkee et al., 2016; Park, Hong, Park, Ha, & Yoo, 2013; Wu, Cheung, Ku, & Hung, 2013). The majority of these negative outcomes could be seen, at least partly, as the results of deficient self-regulation [i.e., high impulsivity, low inhibitory control, and poor decision-making abilities (Billieux, 2012; Choi et al., 2014)].

In recent years, it has been shown that trait impulsivity is often displayed by Internet addicts (IAs; Lee et al., 2012; Yau, Potenza, & White, 2012). Indeed, a large body of research have investigated the role of individual differences in self-regulation, such as trait impulsivity (Chen et al., 2015; Park, Han, & Roh, 2017), among the various psychological factors that potentially lead to excessive use and addiction. Shapira et al. (2003) suggested that IA should be conceptualized as an impulse control disorder. This has been supported by behavioral studies using tasks related to impulse control (Billieux, 2012). For instance, IAs scored higher than controls on the failure to inhibit responses of the GoStop Impulsivity Paradigm (Cao, Su, Liu, & Gao, 2007). Moreover, in a gambling task, IAs selected significantly fewer net decks, and were slower at selecting a strategy (Sun et al., 2009). Nonetheless, IA is often categorized as an addiction, and trait impulsivity has been shown to play a crucial role in developing and maintaining such behavior (Dawe & Loxton, 2004; Schulte, Grilo, & Gearhardt, 2016). Hence, for a better understanding of IA, it is crucial to explore the underlying mechanisms and cortical correlates.

It has been shown that trait impulsivity is related to multiple cortical regions in healthy subjects (Muhlert & Lawrence, 2015; Schilling et al., 2012) and also in several substance addictions (Crews & Boettiger, 2009; Kreek, Nielsen, Butelman, & LaForge, 2005). These studies suggest that the frontal and temporal cortices and the structure of the insula play a crucial role in impulsivity (Du et al., 2016; Matsuo et al., 2009; Moccia et al., 2017) and in the development and maintenance of substance-related addictive behavior. For instance, impulsivity has been shown to have a pivotal role in the onset of and craving for smoking – one of the most common substance addictions. Thus, smokers as a broadly available group in young adults could serve well as a control group in assessing the general features of impulsivity in addictions and the specific influences of impulsivity in IA. Furthermore, other recent studies (Jaworska et al., 2017; Kaag et al., 2014) also emphasize the relevance of the reduction of the cortical surface for impulsiveness-related disorders such as substance use. Smoking, for instance, could lead to overall cortical thinning (Karama et al., 2015) – especially in the frontal regions and the cingulum (Almeida et al., 2008) – and gray matter reduction in the frontal and temporal cortices (Gallinat et al., 2006). These results indicate that the progressive thinning of multiple cortical regions could also play an important role in mediating greater impulsivity in IA.

To this date, to our knowledge, no studies have directly investigated the relationship between cortical thickness and trait impulsivity in IAs compared to healthy controls (HCs). This study aims to fill this gap by evaluating the distinct relationships between trait impulsivity and cortical thickness of the gray matter regions related to addictive behavior in IAs and controls. We tested group differences and group by impulsivity interactions regarding the frontal and temporal cortices and the insula. We hypothesized that regions that show a similar distinct relationship in the two addictive groups (smokers and IAs) compared to HCs would tap into the general effects of impulsivity, whereas regions that occur only for IAs would be the result of the overlap of two different mechanisms – impulsivity and the special characteristics of IA.

## Methods

### Participants

Thirty IAs (15 males and 15 females), 30 age- and gender-matched (15 males and 15 females) HCs, and 30 age- and gender-matched (15 males and 15 females) impulsive control group subjects [smokers, all young adults aged 19–28 years; see Table 1 for mean age and standard deviation (*SD*)] were recruited through the Internet, using university mailing lists. IAs were identified according to the Problematic Internet Use Questionnaire (PIUQ; Demetrovics, Szeredi, & Rózsa, 2008); see further details below. Inclusion criteria for the impulsive control group were smoking at least five cigarettes a day and scoring more than four points (moderate dependence) in the Fagerström Test for Nicotine Dependence (FTND; Fagerstrom & Schneider, 1989; Prokhorov, Pallonen, Fava, Ding, & Niaura, 1996). None of the HC group or the IA members reported smoking. One Internet addict and one control were excluded because they reached the clinical cut-off on the Beck Depression Inventory (BDI), and one more Internet addict was excluded because of failure to understand the questionnaire instructions. Five smokers were also excluded: three of them did not meet our a priori criteria on the FTND, one reached the clinical cut-off on the BDI, and one reached the clinical cut-off on the State and Trait Anxiety Inventory (STAI).

**Table 1.** Sample characteristics of healthy controls (HCs), Internet addicts (IAs), and smokers

	HCs (*n* = 29)	IAs (*n* = 28)	Smokers (*n* = 25)	Differences*
Age	21.86 ± 2.05	21.93 ± 2.09	23.12 ± 3.13	n.s.
Trait impulsivity (BIS)	55.52 ± 9.79	67.30 ± 9.18	63.68 ± 10.28	Smokers and IAs > HCs
Internet addiction (PIUQ)	23.70 ± 2.49	47.73 ± 5.64	23.38 ± 11.04	IAs > HCs and smokers
Depression (BDI)	4.97 ± 5.68	11.13 ± 7.17	8.33 ± 7.42	IAs > smokers > HCs
Anxiety (STAI)	34.60 ± 10.19	45.53 ± 10.51	38.88 ± 11.22	IAs > HCs and smokers
Fragerstörm	–	–	5.65 ± 1.07	–

*Note.* Data are presented as means ± standard deviations where appropriate. BIS: Barratt Impulsiveness Scale; PIUQ: Problematic Internet Use Questionnaire; BDI: Beck Depression Inventory; STAI: State and Trait Anxiety Inventory.

*Differences were considered significant at *p* < .05 with pairwise Bonferroni corrections.

All of the participants were right-handed Caucasian individuals and had a normal body mass index. None of the participants reported alcohol, or other substance- or behavior-related addiction; there were no cross-addictions. Exclusion criteria were any major medical, psychiatric, or neurological disorders.

### Assessments

IA was documented prior to inclusion in the study, using the PIUQ (Demetrovics et al., 2008). The questionnaire consists of 18 items and 3 subscales: obsession, neglect, and control disorder. All items are answered on a 5-point Likert-type scale (*never*, *rarely*, *sometimes*, *often*, and *always*). The three subscales add up to the total score, where a higher score signifies a higher level of addiction. The test has excellent psychometric properties, with a Cronbach’s α of .87 (Demetrovics et al., 2008). In this study, we used the total score. The inclusion criterion for IA was a minimum of 41 points, based on the cut-off suggested by the original authors of the questionnaire (Demetrovics et al., 2008).

The Barratt Impulsiveness Scale (BIS-11; Patton, Stanford, & Barratt, 1995) was used to assess trait impulsivity. The scale consists of 30 items; all items are answered on a 4-point Likert-type scale (*rarely/never*, *occasionally*, *often*, and *almost always/always*). The items add up to the total score, where a higher score signifies higher impulsivity. As in previous studies (Du et al., 2016; Kaag et al., 2014; Tu, Kuan, Li, & Su, 2017), the total score for BIS-11 was used in this study for statistical testing. The BIS-11 has excellent psychometric properties (Cronbach’s α is greater than .8; Patton et al., 1995).

We also assessed individual levels of depression and anxiety. Depression was measured using the BDI (Beck & Steer, 1984), while STAI (Spielberger, 2010) served as an indicator of anxiety. Handedness was measured using the Edinburgh Handedness Inventory (Oldfield, 1971). The FTND (Fagerstrom & Schneider, 1989; Prokhorov et al., 1996) was also administered to the group of smokers to test for nicotine dependence. To exclude possible neurological and psychiatric disorders, substance abuse, and chronic illnesses, the subjects also completed an exploratory questionnaire about lifestyle factors and mental and physical health.

### MRI data collection and analysis

MRI measurements were performed on a 3 Tesla MR scanner (Siemens Magnetom Trio Tim System, Siemens AG, Erlangen, Germany) with a 12-channel head coil. For the volumetric analysis, isotopic 3D T1-weighted sagittal magnetization-prepared rapid acquisition with gradient echo images were used: FOV = 256 × 256 mm^2^, TR = 2,530 ms, TE = 3.37 ms, TI = 1,100 ms, slice thickness = 1 mm, slice number = 176, FA = 7°, bandwidth = 200 Hz/pixel, 256 × 256 matrix.

Freesurfer v6.0 was used for the cortical reconstruction and volumetric segmentation of the images (http://surfer.nmr.mgh.harvard.edu/). Freesurfer software is one of the most reliable automated brain segmentation methods for cortical and subcortical structures. It also allows us to assess the volume of predefined brain structures in a large number of subjects (Fischl et al., 2004; Pardoe, Pell, Abbott, & Jackson, 2009). Freesurfer’s semi-automatic anatomical processing scripts (autorecon 1, 2, and 3) were executed on all the subjects’ data. Visual verifications were performed for all the subjects, and error corrections were applied when indicated.

On the basis of previous studies concerning the relationship between impulsivity and cortical measures in addictions (Du et al., 2016; Limbrick-Oldfield, Van Holst, & Clark, 2013; Matsuo et al., 2009; Moccia et al., 2017), we defined 15 regions of interest (ROIs) using the Desikan–Killiany–Tourville atlas (Klein & Tourville, 2012) implemented in Freesurfer. The ROIs were the left and right frontal cortex [superior frontal cortex, pars opercularis, pars triangularis and pars orbitalis (POrb), the caudal and rostral middle frontal cortex, the lateral and medial orbitofrontal cortex, and the caudal and rostral anterior cingulate cortex (aCC)], the temporal cortex (the inferior-, middle-, and superior temporal cortices and the transverse temporal gyrus), and the insula. The cortical thickness of these predefined cortical ROIs and the total intracranial volume (ICV) was calculated for each participant. Left and right ROIs were entered separately into the analyses.

### Statistical analyses

Data analysis was performed using IBM SPSS Statistics for Windows, version 22.0 (IBM Corp., Armonk, NY, USA). First, we assessed group differences in demographic and clinical characteristics using one-way analyses of variance (ANOVAs). A *p* value less than .05 was considered statistically significant. ANOVAs for the sample characteristics were followed up by Bonferroni corrected pairwise comparisons. After the *p* value, the partial η^2^ values were reported. IAs and smokers were compared to HCs separately. The sections “Results” and “Discussion” focus on IA.

We tested the effect of impulsivity on cortical thickness using an analysis of covariance (ANCOVA). The cortical thicknesses of the ROIs were entered as dependent variables, the group as the independent variable, and the BIS-11 score as a covariate in the ANCOVA. Furthermore, in accordance with previous studies (Perlaki et al., 2014) and to avoid possible confounding effects, the ICV, BDI, and STAI were also included as nuisance covariates. To test whether the correlation between trait impulsivity and the cortical thickness of the ROIs differed between HCs and IAs, the impulsivity by group interaction was also modeled in a separate analysis. We conducted similar ANCOVAs comparing smokers and HCs. The Benjamini–Hochberg (Benjamini & Hochberg, 1995; Glickman, Rao, & Schultz, 2014; Verhoeven, Simonsen, & McIntyre, 2005) false discovery rate (BH FDR) procedure was used to correct for multiple comparison.

Significant main effects and interactions were followed by partial correlations (correcting for ICV, BDI, and STAI), the latter conducted separately for the two groups to test for the direction of the correlation between cortical thickness and trait impulsivity. The significance value of a correlation analysis is highly dependent on the sample size, such that the probability of type II error increases when the sample size is low (Ellis, 2010). Therefore, the advice in such cases is to interpret results based on the effect size of the correlation. Since our sample size was relatively small (the control group consisted of 29 people and the IA group of 28), we focused on correlation coefficients rather than *p* values. An *r* value of .1 was considered as weak, a coefficient of .3 as medium, and a coefficient of .5 as strong correlation between the variables (Cohen, 1992).

### Ethics

The research was approved by the local ethical review committee and was carried out in accordance with the Code of Ethics of the World Medical Association (Declaration of Helsinki); informed consent was obtained from all of the participants involved in the study.

## Results

### Sample characteristics

The BIS-11 scores were significantly higher in IAs and smokers than in the controls (*F*_2, 79_ = 11.001, *p* < .01, η^2^_p_ = 0.22), while IAs scored higher on the PIUQ than the two control groups (*F*_2, 79_ = 49.962, *p* < .01, η^2^_p_ = 0.57). Regarding BDI, IAs achieved higher scores than the two control groups, who also differed, with smokers scoring higher than HCs (*F*_2, 79_ = 5.763, *p* = .01, η^2^_p_ = 0.13). IAs reported higher levels of anxiety (STAI) than the two control groups (*F*_2, 79_ = 8.073, *p* < .01, η^2^_p_ = 0.17). Table 1 shows the mean scores, *SD*s, and group differences.

### Cortical thickness comparison between groups

The thickness of the left superior temporal cortex (STC; *F*_1, 52_ = 6.578, *p* = .013, η^2^_p_ = 0.11) was significantly greater in the HC group (*M* = 2.884, *SD* = 0.117) than in the IA group (*M* = 2.825, *SD* = 0.108). The between-group comparisons of thicknesses of the other ROIs were not significant. Table 2 depicts the mean thicknesses, *SD*s, and statistical results between IAs and HCs. Similarly, the thickness of the bilateral STC (left: *F*_1, 47_ = 4.576, *p* = .041, η^2^_p_ = 0.09; right: *F*_1, 47_ = 6.002, *p* = .018, η^2^_p_ = 0.11) was significantly greater in HCs than in smokers (left: *M* = 2.805, *SD* = 0.141). However, the left STC did not survive the BH FDR correction. Table 3 depicts the mean thicknesses, *SD*s, and statistical results for the comparison between HCs and smokers.

**Table 2.** Between- and within-group MANCOVA and post-hoc partial correlation results between IAs and HCs

Name of ROI	Group main effect (ANCOVA)^a^					Impulsivity main effect (ANCOVA)^a^				Group by impulsivity interaction (ANCOVA)^a^				
	Size^b^		Statistics			Statistics				Statistics				
	HCs	IAs	*F*	*p*	η^2^	*F*	*p*	η^2^	*r*^b,c^	*F*	*p*	η^2^	*r* (HCs)^b,d^	*r* (IAs)^b,d^
Left superior frontal cortex	2.799 ± 0.118	2.795 ± 0.092	*F*_1, 52_ = 0.023	n.s.	0.00	*F*_1, 51_ = 0.169	n.s.	0.00		*F*_1, 50_ = 0.270	n.s.	0.00		
Left pars opercularis	2.648 ± 0.099	2.681 ± 0.116	*F*_1, 52_ = 0.708	n.s.	0.01	*F*_1, 51_ = 0.577	n.s.	0.01		*F*_1, 50_ = 0.047	n.s.	0.00		
Left pars triangularis	2.524 ± 0.098	2.530 ± 0.117	*F*_1, 52_ = 0.663	n.s.	0.01	*F*_1, 51_ = 0.169	n.s.	0.01		*F*_1, 50_ = 0.163	n.s.	0.00		
Left pars orbitalis	2.729 ± 0.144	2.749 ± 0.128	*F*_1, 52_ = 0.689	n.s.	0.01	*F*_1, 51_ = 8.429	.005*	0.14	−.29	*F*_1, 50_ = 0.253	n.s.	0.00		
Left caudal middle frontal cortex	2.637 ± 0.125	2.642 ± 0.082	*F*_1, 52_ = 0.796	n.s.	0.01	*F*_1, 51_ = 0.273	n.s.	0.00		*F*_1, 50_ = 0.503	n.s.	0.01		
Left rostal middle frontal cortex	2.446 ± 0.107	2.452 ± 0.080	*F*_1, 52_ = 0.036	n.s.	0.00	*F*_1, 51_ = 0.333	n.s.	0.01		*F*_1, 50_ = 0.003	n.s.	0.00		
Left lateral orbitofrontal cortex	2.709 ± 0.093	2.713 ± 0.091	*F*_1, 52_ = 0.110	n.s.	0.00	*F*_1, 51_ = 0.081	n.s.	0.00		*F*_1, 50_ = 0.659	n.s.	0.01		
Left medial orbitofrontal cortex	2.454 ± 0.092	2.467 ± 0.105	*F*_1, 52_ = 0.928	n.s.	0.02	*F*_1, 51_ = 0.001	n.s.	0.00		*F*_1, 50_ = 0.024	n.s.	0.00		
Left caudal anterior cingulate cortex	2.717 ± 0.206	2.656 ± 0.215	*F*_1, 52_ = 0.217	n.s.	0.00	*F*_1, 51_ = 2.156	n.s.	0.04		*F*_1, 50_ = 2.650	n.s.	0.05		
Left rostal anterior cingulat e cortex	2.911 ± 0.156	2.861 ± 0.125	*F*_1, 52_ = 2.045	n.s.	0.04	*F*_1, 51_ = 1.093	n.s.	0.02		*F*_1, 50_ = 1.071	n.s.	0.02		
Left superior temporal cortex	2.884 ± 0.117	2.825 ± 0.108	*F*_1, 52_ = 6.578	.013*	0.11	*F*_1, 51_ = 0.063	n.s.	0.00		*F*_1, 50_ = 3.236	n.s.	0.06		
Left middle temporal cortex	2.840 ± 0.105	2.842 ± 0.123	*F*_1, 52_ = 0.001	n.s.	0.00	*F*_1, 51_ = 0.053	n.s.	0.00		*F*_1, 50_ = 6.553	.014*	0.12	.35	−.35
Left inferior temporal cortex	2.752 ± 0.143	2.736 ± 0.154	*F*_1, 52_ = 0.012	n.s.	0.00	*F*_1, 51_ = 0.178	n.s.	0.00		*F*_1, 50_ = 4.126	.048**	0.08	.29	−.26
Left transverse temporal gyrus	2.605 ± 0.039	2.502 ± 0.040	*F*_1, 52_ = 1.900	n.s.	0.03	*F*_1, 51_ = 0.059	n.s.	0.00		*F*_1, 50_ = 6.738	.012*	0.12	.32	−.34
Left insula	3.062 ± 0.076	3.077 ± 0.109	*F*_1 ,52_ = 0.228	n.s.	0.00	*F*_1, 51_ = 5.435	.024*	0.09	.32	*F*_1, 50_ = 3.129	n.s.	0.06		
Right superior frontal cortex	2.748 ± 0.109	2.733 ± 0.098	*F*_1, 52_ = 0.006	n.s.	0.00	*F*_1, 51_ = 0.022	n.s.	0.00		*F*_1, 50_ = 0.614	n.s.	0.01		
Right pars opercularis	2.626 ± 0.080	2.658 ± 0.127	*F*_1, 52_ = 2.448	n.s.	0.04	*F*_1, 51_ = 0.438	n.s.	0.01		*F*_1, 50_ = 0.159	n.s.	0.00		
Right pars triangularis	2.500 ± 0.094	2.525 ± 0.100	*F*_1, 52_ = 0.712	n.s.	0.01	*F*_1, 51_ = 1.676	n.s.	0.03		*F*_1, 50_ = 1.503	n.s.	0.03		
Right pars orbitalis	2.643 ± 0.138	2.708 ± 0.154	*F*_1, 52_ = 2.002	n.s.	0.04	*F*_1, 51_ = 0.270	n.s.	0.00		*F*_1, 50_ = 0.037	n.s.	0.00		
Right caudal middle frontal cortex	2.605 ± 0.112	2.630 ± 0.072	*F*_1, 52_ = 1.971	n.s.	0.04	*F*_1, 51_ = 0.000	n.s.	0.00		*F*_1, 50_ = 0.149	n.s.	0.00		
Right rostal middle frontal cortex	2.366 ± 0.082	2.355 ± 0.075	*F*_1, 52_ = 0.010	n.s.	0.00	*F*_1, 51_ = 0.178	n.s.	0.00		*F*_1, 50_ = 2.178	n.s.	0.04		
Right lateral orbitofrontal cortex	2.603 ± 0.095	2.607 ± 0.123	*F*_1, 52_ = 0.473	n.s.	0.01	*F*_1, 51_ = 1.905	n.s.	0.04		*F*_1, 50_ = 0.034	n.s.	0.00		
Right medial orbitofrontal cortex	2.434 ± 0.129	2.430 ± 0.110	*F*_1, 52_ = 0.217	n.s.	0.00	*F*_1, 51_ = 1.450	n.s.	0.03		*F*_1, 50_ = 0.008	n.s.	0.00		
Right caudal anterior cingulate cortex	2.488 ± 0.183	2.492 ± 0.177	*F*_1, 52_ = 0.142	n.s.	0.00	*F*_1, 51_ = 0.884	n.s.	0.02		*F*_1, 50_ = 0.088	n.s.	0.00		
Right rostal anterior cingulate cortex	2.827 ± 0.156	2.838 ± 0.164	*F*_1, 52_ = 0.672	n.s.	0.01	*F*_1, 51_ = 0.580	n.s.	0.01		*F*_1, 50_ = 0.053	n.s.	0.00		
Right superior temporal cortex	2.908 ± 0.130	2.876 ± 0.105	*F*_1, 52_ = 1.120	n.s.	0.02	*F*_1, 51_ = 1.224	n.s.	0.02		*F*_1, 50_ = 4.176	.046**	0.08	.35	−.21
Right middle temporal cortex	2.936 ± 0.106	2.912 ± 0.111	*F*_1, 52_ = 0.058	n.s.	0.00	*F*_1, 51_ = 0.649	n.s.	0.01		*F*_1, 50_ = 6.099	.017*	0.11	.39	−.25
Right inferior temporal cortex	2.832 ± 0.076	2.814 ± 0.107	*F*_1, 52_ = 0.009	n.s.	0.00	*F*_1, 51_ = 0.139	n.s.	0.00		*F*_1, 50_ = 2.135	n.s.	0.04		
Right transverse temporal gyrus	2.532 ± 0.155	2.504 ± 0.149	*F*_1, 52_ = 0.739	n.s.	0.01	*F*_1, 51_ = 0.167	n.s.	0.00		*F*_1, 50_ = 0.000	n.s.	0.00		
Right insula	3.107 ± 0.089	3.091 ± 0.091	*F*_1, 52_ = 0.506	n.s.	0.01	*F*_1, 51_ = 11.425	.001*	0.18	.41	*F*_1, 50_ = 0.188	n.s.	0.00		

*Note.* MANCOVA: multivariate analysis of covariance; HC: healthy control; IAs: Internet addicts; ANCOVA: analysis of covariance; ROI: region of interest.

^a^Adjusted for ICV, BDI, and STAI. ^b^Values represent means ± standard deviations. ^c^Values represent Pearson’s partial correlation coefficients between CT and impulsivity (BIS-11 total score) for IAs and HCs together. ^d^Values represent Pearson’s partial correlation coefficients between CT and impulsivity (BIS-11 total score).

*Significant *p* value Benjamini–Hochberg adjusted for multiple comparisons. **Did not survive Benjamini–Hochberg correction.

**Table 3.** Between- and within-group MANCOVA and post-hoc partial correlation results between smokers and HCs

Name of ROI	Group main effect (ANCOVA)^a^					Impulsivity main effect (ANCOVA)^a^				Group by impulsivity interaction (ANCOVA)^a^				
	Size^b^		Statistics			Statistics				Statistics				
	HCs	Smokers	*F*	*p*	η^2^	*F*	*p*	η^2^	*r*^b,c^	*F*	*p*	η^2^	*r* (HCs)^b,d^	*r* (IAs)^b,d^
Left superior frontal cortex	2.799 ± 0.118	2.725 ± 0.124	*F*_1, 47_ = 3.767	n.s.	0.07	*F*_1, 47_ = 0.060	n.s.	0.00		*F*_1, 46_ = 0.994	n.s.	0.02		
Left pars opercularis	2.648 ± 0.099	2.612 ± 0.111	*F*_1, 47_ = 0.955	n.s.	0.02	*F*_1, 47_ = 0.037	n.s.	0.00		*F*_1, 46_ = 0.014	n.s.	0.00		
Left pars triangularis	2.524 ± 0.098	2.471 ± 0.129	*F*_1, 47_ = 0.606	n.s.	0.01	*F*_1, 47_ = 0.692	n.s.	0.02		*F*_1, 46_ = 2.089	n.s.	0.04		
Left pars orbitalis	2.729 ± 0.144	2.689 ± 0.097	*F*_1, 47_ = 0.459	n.s.	0.01	*F*_1, 47_ = 2.004	n.s.	0.02		*F*_1, 46_ = 1.316	n.s.	0.03		
Left caudal middle frontal cortex	2.637 ± 0.125	2.606 ± 0.123	*F*_1, 47_ = 0.062	n.s.	0.00	*F*_1, 47_ = 0.045	n.s.	0.00		*F*_1, 46_ = 2.629	n.s.	0.05		
Left rostal middle frontal cortex	2.446 ± 0.107	2.392 ± 0.118	*F*_1, 47_ = 2.334	n.s.	0.05	*F*_1, 47_ = 0.248	n.s.	0.01		*F*_1, 46_ = 1.171	n.s.	0.03		
Left lateral orbitofrontal cortex	2.709 ± 0.093	2.657 ± 0.118	*F*_1, 47_ = 3.334	n.s.	0.07	*F*_1, 47_ = 0.006	n.s.	0.00		*F*_1, 46_ = 0.394	n.s.	0.01		
Left medial orbitofrontal cortex	2.454 ± 0.092	2.423 ± 0.122	*F*_1, 47_ = 1.021	n.s.	0.02	*F*_1, 47_ = 0.105	n.s.	0.00		*F*_1, 46_ = 0.146	n.s.	0.00		
Left caudal anterior cingulate cortex	2.717 ± 0.206	2.603 ± 0.154	*F*_1, 47_ = 9.838	.003*	0.17	*F*_1, 47_ = 5.376	.025*	0.10	.245	*F*_1, 46_ = 1.032	n.s.	0.02		
Left rostal anterior cingulate cortex	2.911 ± 0.156	2.837 ± 0.188	*F*_1, 47_ = 4.593	.037**	0.09	*F*_1, 47_ = 0.002	n.s.	0.00		*F*_1, 46_ = 0.040	n.s.	0.00		
Left superior temporal cortex	2.884 ± 0.117	2.805 ± 0.141	*F*_1, 47_ = 4.576	.041**	0.09	*F*_1, 47_ = 0.082	n.s.	0.00		*F*_1, 46_ = 2.705	n.s.	0.03		
Left middle temporal cortex	2.840 ± 0.105	2.845 ± 0.147	*F*_1, 47_ = 0.012	n.s.	0.00	*F*_1, 47_ = 0.454	n.s.	0.01		*F*_1, 46_ = 4.372	.04**	0.09	.35	−.17
Left inferior temporal cortex	2.752 ± 0.143	2.699 ± 0.156	*F*_1, 47_ = 1.342	n.s.	0.03	*F*_1, 47_ = 0.739	n.s.	0.02		*F*_1, 46_ = 1.556	n.s.	0.03		
Left transverse temporal gyrus	2.605 ± 0.039	2.497 ± 0.133	*F*_1, 47_ = 9.806	.003*	0.17	*F*_1, 47_ = 3.032	n.s.	0.06		*F*_1, 46_ = 0.925	n.s.	0.03		
Left insula	3.062 ± 0.076	2.977 ± 0.162	*F*_1, 47_ = 0.929	n.s.	0.02	*F*_1, 47_ = 2.108	n.s.	0.04		*F*_1, 46_ = 0.936	n.s.	0.03		
Right superior frontal cortex	2.748 ± 0.109	2.676 ± 0.112	*F*_1, 47_ = 3.641	n.s.	0.07	*F*_1, 47_ = 0.189	n.s.	0.00		*F*_1, 46_ = 1.214	n.s.	0.03		
Right pars opercularis	2.626 ± 0.080	2.618 ± 0.151	*F*_1, 47_ = 0.008	n.s.	0.00	*F*_1, 47_ = 0.453	n.s.	0.01		*F*_1, 46_ = 0.001	n.s.	0.00		
Right pars triangularis	2.500 ± 0.094	2.451 ± 0.103	*F*_1, 47_ = 1.730	n.s.	0.04	*F*_1, 47_ = 1.986	n.s.	0.04		*F*_1, 46_ = 0.342	n.s.	0.01		
Right pars orbitalis	2.643 ± 0.138	2.626 ± 0.127	*F*_1, 47_ = 0.794	n.s.	0.02	*F*_1, 47_ = 0.022	n.s.	0.00		*F*_1, 46_ = 0.160	n.s.	0.00		
Right caudal middle frontal cortex	2.605 ± 0.112	2.586 ± 0.117	*F*_1, 47_ = 0.010	n.s.	0.00	*F*_1, 47_ = 0.175	n.s.	0.00		*F*_1, 46_ = 0.188	n.s.	0.00		
Right rostal middle frontal cortex	2.366 ± 0.082	2.313 ± 0.089	*F*_1, 47_ = 4.385	.042**	0.09	*F*_1, 47_ = 0.131	n.s.	0.00		*F*_1, 46_ = 1.705	n.s.	0.04		
Right lateral orbitofrontal cortex	2.603 ± 0.095	2.583 ± 0.125	*F*_1, 47_ = 1.174	n.s.	0.04	*F*_1, 47_ = 0.329	n.s.	0.01		*F*_1, 46_ = 1.991	n.s.	0.04		
Right medial orbitofrontal cortex	2.434 ± 0.129	2.364 ± 0.121	*F*_1, 47_ = 2.683	n.s.	0.05	*F*_1, 47_ = 0.022	n.s.	0.00		*F*_1, 46_ = 0.825	n.s.	0.02		
Right caudal anterior cingulate cortex	2.488 ± 0.183	2.428 ± 0.094	*F*_1, 47_ = 1.425	n.s.	0.03	*F*_1, 47_ = 0.067	n.s.	0.00		*F*_1, 46_ = 0.157	n.s.	0.00		
Right rostal anterior cingulate cortex	2.827 ± 0.156	2.734 ± 0.159	*F*_1, 47_ = 3.620	n.s.	0.07	*F*_1, 47_ = 0.044	n.s.	0.00		*F*_1, 46_ = 0.138	n.s.	0.00		
Right superior temporal cortex	2.908 ± 0.130	2.827 ± 0.133	*F*_1, 47_ = 6.002	.018*	0.11	*F*_1, 47_ = 1.392	n.s.	0.03		*F*_1, 46_ = 9.010	.001*	0.16	.35	−.33
Right middle temporal cortex	2.936 ± 0.106	2.871 ± 0.119	*F*_1, 47_ = 3.050	n.s.	0.06	*F*_1, 47_ = 0.470	n.s.	0.01		*F*_1, 46_ = 10.622	.01*	0.18	.39	−.39
Right inferior temporal cortex	2.832 ± 0.076	2.800 ± 0.145	*F*_1, 47_ = 0.272	n.s.	0.01	*F*_1, 47_ = 0.001	n.s.	0.00		*F*_1, 46_ = 0.086	n.s.	0.00		
Right transverse temporal gyrus	2.532 ± 0.155	2.566 ± 0.210	*F*_1, 47_ = 0.002	n.s.	0.00	*F*_1, 47_ = 0.502	n.s.	0.01		*F*_1, 46_ = 2.035	n.s.	0.04		
Right insula	3.107 ± 0.089	2.987 ± 0.163	*F*_1, 47_ = 0.983	n.s.	0.02	*F*_1, 47_ = 1.596	n.s.	0.03		*F*_1, 46_ = 0.533	n.s.	0.01		

*Note.* MANCOVA: multivariate analysis of covariance; HCs: healthy controls; IAs: Internet addicts; ANCOVA: analysis of covariance; ROI: region of interest.

^a^Adjusted for ICV, BDI, and STAI. ^b^Values represent means ± standard deviations. ^c^Values represent Pearson’s partial correlation coefficients between CT and impulsivity (BIS-11 total score) for HCs and smokers together. ^d^Values represent Pearson’s partial correlation coefficients between CT and impulsivity (BIS-11 total score).

*Significant *p* value Benjamini–Hochberg adjusted for multiple comparisons. **Did not survive Benjamini–Hochberg correction.

### Relationship between cortical thickness and trait impulsivity

The ANCOVAs revealed that impulsivity has a significant main effect on two ROIs: the left POrb (*F*_1, 51_ = 8.429, *p* = .005, η^2^_p_ = 0.14), and the bilateral insula (right: *F*_1, 51_ = 11.425, *p* = .001, η^2^_p_ = 0.18; left: *F*_1, 51_ = 5.435, *p* = .024, η^2^_p_ = 0.09). The within-group comparisons of the thicknesses of the other ROIs were not significant.

The follow-up partial correlation analyses showed that a higher level of impulsivity is associated with a thinner cortex in the left POrb (*r* = −.29). Furthermore, trait impulsivity and cortical thickness of both the right (*r* = .41) and the left (*r* = .32) insula are positively correlated. Table 2 demonstrates all the ANCOVAs and post-hoc test statistics.

### Relationship between cortical thickness and addiction severity

To see whether addiction severity had any further effect on the cortical thickness of the ROIs, Pearson’s correlations were used. For the IA group, the correlation analyses revealed only one significant relationship between the cortical thickness of the left rostral aCC and the PIUQ total score (*r* = .4, *p* = .03). This result did not survive the HB FDR correction for multiple comparisons. For the smokers, we did not find a significant relationship between the cortical thickness of the ROIs and the Fagerström scores. Supplementary Table 1 demonstrates all the correlational test statistics.

### Relationship between cortical thickness and trait impulsivity between groups

The ANCOVAs showed a group by impulsivity interaction across the following ROIs: right STC (*F*_1, 50_ = 4.176, *p* = .05, η^2^_p_ = 0.08), bilateral middle temporal cortex (MTC; left: *F*_1, 50_ = 6.553, *p* = .01, η^2^_p_ = 0.12; right: *F*_1, 50_ = 6.099, *p* = .02, η^2^_p_ = 0.11), left inferior temporal cortex (ITC; *F*_1, 50_ = 4.126, *p* = .05, η^2^_p_ = 0.08), and left transverse temporal cortex (TTC; *F*_1, 50_ = 6.738, *p* = .01, η^2^_p_ = 0.12). However, the right STC and left ITC interactions did not survive the BH FDR correction. Table 2 shows all within-group ANCOVA test statistics.

**Figure 1.**
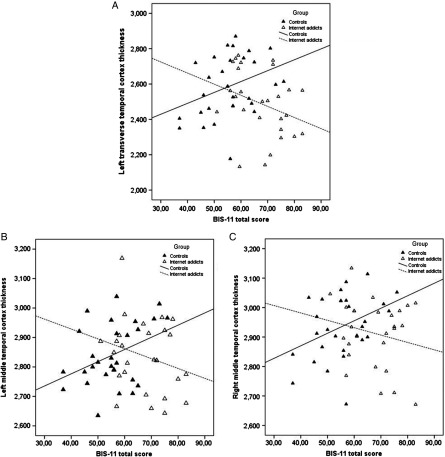
Correlations between BIS-11 total scores and cortical thickness measures. There was a significant interaction between the total score of the Barrat Impulsiveness Scale (BIS-11) and group in cortical thicknesses of the left transverse temporal cortex (panel A: *r* = .32 for controls; *r* = −.34 for Internet addicts), cortical thickness of the left middle temporal cortex (panel B: *r* = .35 for controls; *r* = −.35 for Internet addicts), and cortical thickness of the right middle temporal cortex (panel C: *r* = .39 for controls; *r* = −.25 for Internet addicts). Please note that the cortical thickness values displayed here are uncorrected for covariates

The post-hoc partial correlation analyses revealed positive relationships with medium power between cortical thickness and impulsivity across all four ROIs in HCs: left MTC *r* = .35, left TTC *r* = .32, and right MTC *r* = .39. As expected, in the IA group, the correlation coefficients were found to be negative. The correlation between the left MTC (*r* = −.35) and left TTC (*r* = −.34) and impulsivity had medium power, whereas for the right MTC (*r* = −.25), it had weak power. Table 2 depicts the correlation coefficients and Figure 1 shows group-wise comparisons.

We conducted similar ANCOVAs comparing smokers and HCs, including only the ROIs that showed significant interaction when comparing IAs and HCs. We found significant interaction in the right STC (right: *F*_1, 47_ = 9.01, *p* < .01, η^2^_p_ = 0.16) and bilateral MTC (left: *F*_1, 47_ = 4.372, *p* = .04, η^2^_p_ = 0.09; right: *F*_1, 47_ = 10.622, *p* < .01, η^2^_p_ = 0.18), but not in the left ITC (*F*_1, 47_ = 1.556, *p* = .22, η^2^_p_ = 0.03) or the left TTC (*F*_1, 47_ = 0.925, *p* = .34, η^2^_p_ = 0.03). Furthermore, the left MTC correlation did not survive the BH FDR correction.

In this study, post-hoc correlations revealed that, concerning the IA group, the correlation coefficients were negative. The correlation between the right STC (*r* = −.33) and the right MTC (*r* = −.39) had medium power.

## Discussion

### Summary

To our knowledge, this is the first study investigating the relationship between impulsivity and cortical thickness in IAs. The IAs and controls differed in the cortical thickness of the left STC. Moreover, there was a main effect of impulsivity on cortical morphology observed in the left POrb and bilateral insula. In addition, we identified distinct relationships between impulsivity and cortical thickness in IAs and controls regarding the left TTC and bilateral MTC.

### Group differences

As in our previous study (Altbäcker et al., 2016) and those of others (Park et al., 2017; Yuan et al., 2013), we found differences between IAs and controls. The left STC was found to be thicker for controls. The medial temporal cortex is associated with urges and craving (Ko et al., 2009) and, along with the STC, is involved in regulatory control over reward-seeking behavior (Chiamulera, 2005). This is consistent with our results: the changes in the cortical thickness of the left STC were found to be significant when comparing the addiction groups (IAs and smokers) and the HCs. Furthermore, the present research suggests that the differences between IAs and HCs are already present in young adults.

It should also be noted that we found additional group differences between smokers and HCs, the most prominent being in the left caudal aCC. This difference might be due to the fact that smoking is a substance-related addiction, whereas IA is not. Nevertheless, previous studies (Lee, Park, Namkoong, Kim, & Jung, 2018; Van Rooij & Prause, 2014) have shown similar cortical changes of the aCC in IA.

### The relationship between impulsivity and cortical thickness

Impulsivity had a significant main effect, regardless of group, on the left POrb and the bilateral insula. The post-hoc correlation revealed a negative relationship between the thickness of the POrb and impulsivity. Interestingly, trait impulsivity was positively correlated with the thickness of the insula. There are pieces of evidence suggesting that the left IFC (Aron, Robbins, & Poldrack, 2014) and the POrb in particular (Swick, Ashley, & Turken, 2008) are involved in inhibitory mechanisms. In an fMRI study with healthy adults, Xue, Lu, Levin, and Bechara (2010) suggest that activation in the insula signals a gambling urge (Tényi, Gyimesi, Kovács, Tényi, & Janszky, 2016). Moreover, it has been proposed (Brevers, Noël, He, Melrose, & Bechara, 2016; Verdejo-García & Bechara, 2009) that this urge might facilitate the seeking of immediate rewards.

### The distinctive connection between trait impulsivity and cortical thickness in IAs

The distinct relationships between cortical thickness and impulsivity in IAs compared to controls could be identified as either a predisposition to or a consequence of IA, or even a combination of both, as found in patients with major depression (Fradkin, Khadka, Bessette, & Stevens, 2017) and cocaine addicts (Kaag et al., 2014). Consistent with previous studies (Park et al., 2017; Yuan et al., 2013), we found negative correlations in IAs between several cortical areas that are involved in impulsivity and executive functions, such as impaired decision-making and poor response inhibition. The positive correlation in controls is in line with previous studies (Antonucci et al., 2006; Aron, Robbins, & Poldrack, 2004; Schilling et al., 2012). A possible interpretation of the present findings might be that a normal trait impulsivity is essential for intact functioning (Doran, Spring, McChargue, Pergadia, & Richmond, 2004) but that, nevertheless, overly high levels of impulsivity might lead to negative consequences.

Impulsivity has been shown (Crews & Boettiger, 2009; Du et al., 2016; Kreek et al., 2005; Limbrick-Oldfield et al., 2013; Matsuo et al., 2009; Moccia et al., 2017) to be among the most influential underlying factors in developing an addiction of any kind. Moreover, addictive behavior could further increase impulsivity (de Wit, 2009). Furthermore, a large body of research (Altbäcker et al., 2016; Kühn & Gallinat, 2015; Park et al., 2017; Yuan et al., 2013) have shown that IA has an influence on the morphology of several brain regions. Thus, it seems plausible to claim that the effects of impulsivity, combined with long-term exposure to some specific substance or stimuli, might result in different natures of the relationships between impulsivity and brain structure when compared to HCs. It has been shown (Balogh, Mayes, & Potenza, 2013; Blum et al., 2014; Grant, Potenza, Weinstein, & Gorelick, 2010) that substance-related and behavioral addictions often lead to similar morphological or functional changes of the brain. In this study, we found similar interactions in the right STC and MTC when comparing IAs and HCs to those we found when comparing smokers and HCs. A previous study by Kaag et al. (2014) also found the same interaction in cocaine users and HCs in the STC.

Importantly, our results also suggest that the left MTC and TTC might be distinct areas that are unique for IA, as these interactions were not present when comparing smokers and HCs. As previous studies (Ding et al., 2014; Dong, Huang, & Du, 2012; Feng et al., 2013) have claimed, temporal areas are involved in audio–visual processing – a function that is frequently activated during screen media activity like computer and Internet use. It has been suggested that long-term hyperactivity in such regions could lead to impaired auditory–visual abilities (Ding et al., 2014), language-processing impairment (Söderström, Horne, Mannfolk, van Westen, & Roll, 2017), and learning problems (Cone-Wesson, 2005). The negative relationship between impulsivity and the thickness of the left POrb observed in the whole sample could lend further support to the possible involvement of language processing. We argue that this is a possible interpretation of the overlapping networks of impulsivity alterations in cortical thickness. Although the temporal cortex has been shown to be impaired in both chronic drug abuse (Robbins, Ersche, & Everitt, 2008) and IA (Hahn & Kim, 2014; Weinstein & Lejoyeux, 2010), it is still possible that our results are due to the fact that we compared a substance-related to a behavioral addict group. Overall, our findings suggest that changes in cortical thickness in IAs, in a similar way to substance-related addictions, are at least partly mediated by trait impulsivity.

These findings may have clinically relevant implications as well. Previous research has shown that impulsivity plays a crucial role in treatment sensitivity and predicting absence outcomes in, for instance, smoking, marijuana, cocaine, heroin, and methamphetamine addicts (see Stevens et al., 2014 for a review). Our results show that impulsivity is a relevant factor in IA and therefore that it is plausible that, as for other addictions, special focus might be needed during therapy to help individuals overcome this issue. More research is needed to discover the manner in which clinicians might tailor treatment for individuals who are more prone to impulsivity, which may also be connected to the duration of the addiction disorder.

Some limitations of this study should be noted. First, we relied on self-reported measures of IA and impulsivity. Despite this limitation, to date, IA can only be assessed by questionnaires, and the PIUQ has been proved to be a well-established and reliable measure of IA. The BIS-11 is also a widely used and validated measure for trait impulsivity. However, it would be interesting for further studies to include a behavioral measure of impulsivity, as this might lead to more accurate results. Furthermore, the modest sample size may limit the generalization of our findings, as it was particularly noticeable in relation to partial correlations. On the whole, the interaction of impulsivity and excessive Internet use in the context of thickness changes in the temporal cortex is unclear. It may be noted that both impulsivity and Internet overuse independently lead to alterations in the cortical thickness of the temporal cortex, or that the presence of both impulsivity and Internet overuse has a supra-additive effect on cortical thickness, or that cortical thickness changes are primarily driven by impulsivity. Only larger studies will help to disentangle these possibilities.

## Conclusions

Notwithstanding these limitations, our results, in sum, provide additional insights into the etiology of IA and the underlying cortical correlates of impulsive behavior in IAs that are similar to what has been observed in substance-related addictions. This study provides additional evidence of the importance of including measures of cortical thickness for detecting and understanding the origin of cortical abnormalities in IA. Moreover, our results provide further evidence that IA has shared characteristics with other addictive behaviors. Furthermore, the current findings may foreshadow some clinically relevant implications for future treatment, such as developing complex strategies to address IAs with different levels of trait impulsivity. Finally, future studies will be necessary to test these implications in clinical practice to clarify the present findings and to translate them into successful IA prevention and cessation outcomes.

## References

[B1] Aboujaoude E. (2010). Problematic Internet use: An overview. World Psychiatry, 9(2), 85–90. doi:10.1002/j.2051-5545.2010.tb00278.x 20671890 PMC2911081

[B2] Aboujaoude E. (2017). The Internet’s effect on personality traits: An important casualty of the “Internet addiction” paradigm. Journal of Behavioral Addictions, 6(1), 1–4. doi:10.1556/2006.6.2017.009 PMC5573001 28301969

[B3] Almeida O. P. Garrido G. J. Lautenschlager N. T. Hulse G. K. Jamrozik K. Flicker L. (2008). Smoking is associated with reduced cortical regional gray matter density in brain regions associated with incipient Alzheimer disease. The American Journal of Geriatric Psychiatry, 16(1), 92–98. doi:10.1097/JGP.0b013e318157cad2 18165464

[B4] Altbäcker A. Plózer E. Darnai G. Perlaki G. Horváth R. Orsi G. Nagy S. A. Bogner P. Schwarcz A. Kovács N. Komoly S. Clemens Z. Janszky J. (2016). Problematic Internet use is associated with structural alterations in the brain reward system in females. Brain Imaging and Behavior, 10(4), 953–959. doi:10.1007/s11682-015-9454-9 26399236

[B5] Antonucci A. S. Gansler D. A. Tan S. Bhadelia R. Patz S. Fulwiler C. (2006). Orbitofrontal correlates of aggression and impulsivity in psychiatric patients. Psychiatry Research – Neuroimaging, 147(2–3), 213–220. doi:10.1016/j.pscychresns.2005.05.016 16952446

[B6] Aron A. R. Robbins T. W. Poldrack R. A. (2004). Inhibition and the right inferior frontal cortex. Trends in Cognitive Sciences, 8(4), 170–177. doi:10.1016/j.tics.2004.02.010 15050513

[B7] Aron A. R. Robbins T. W. Poldrack R. A. (2014). Inhibition and the right inferior frontal cortex: One decade on. Trends in Cognitive Sciences, 18(4), 177–1855. doi:10.1016/j.tics.2013.12.003 24440116

[B8] Balogh K. N. Mayes L. C. Potenza M. N. (2013). Risk-taking and decision-making in youth: Relationships to addiction vulnerability. Journal of Behavioral Addictions, 2(1), 1–9. doi:10.1556/JBA.2.2013.1.1 24294500 PMC3840427

[B9] Beck A. T. Steer R. A. (1984). Internal consistencies of the original and revised Beck Depression Inventory. Journal of Clinical Psychology, 40(6), 1365–1367. doi:10.1002/1097-4679(198411)40:6<1365::AID-JCLP2270400615>3.0.CO;2-D 6511949

[B10] Benjamini Y. Hochberg Y. (1995). Controlling the false discovery rate: A practical and powerful approach to multiple. Journal of the Royal Statistical Society. Series B (Methodological), 57(1), 289–300. Retrieved from https://pdfs.semanticscholar.org/c508/3a5289fb443f8f61a8685d409de7c8757a4b.pdf

[B11] Billieux J. (2012). Problematic use of the Internet and self-regulation: A review of the initial studies. The Open Addiction Journal, 5(1), 24–29. doi:10.2174/1874941001205010024

[B12] Block J. J. (2008). Issues for DSM-V: Internet addiction. American Journal of Psychiatry, 165(3), 306–307. doi:10.1176/appi.ajp.2007.07101556 18316427

[B13] Blum K. Febo M. Mclaughlin T. Cronjé F. J. Han D. Gold M. S. (2014). Hatching the behavioral addiction egg: Reward Deficiency Solution System (RDSS)^TM^ as a function of dopaminergic neurogenetics and brain functional connectivity linking all addictions under a common rubric. Journal of Behavioral Addictions, 3(3), 149–156. doi:10.1556/JBA.3.2014.019 25317338 PMC4189308

[B14] Brevers D. Noël X. He Q. Melrose J. A. Bechara A. (2016). Increased ventral-striatal activity during monetary decision making is a marker of problem poker gambling severity. Addiction Biology, 21(3), 688–699. doi:10.1111/adb.12239 25781641 PMC4573763

[B15] Cao F. Su L. Liu T. Q. Gao X. (2007). The relationship between impulsivity and Internet addiction in a sample of Chinese adolescents. European Psychiatry, 22(7), 466–471. doi:10.1016/j.eurpsy.2007.05.004 17765486

[B16] Chen C.-Y. Huang M.-F. Yen J.-Y. Chen C.-S. Liu G.-C. Yen C.-F. Ko C.-H. (2015). Brain correlates of response inhibition in Internet gaming disorder. Psychiatry and Clinical Neurosciences, 69(4), 201–209. doi:10.1111/pcn.12224 25047685

[B17] Chiamulera C. (2005). Cue reactivity in nicotine and tobacco dependence: A “multiple-action” model of nicotine as a primary reinforcement and as an enhancer of the effects of smoking-associated stimuli. Brain Research Reviews, 48(1), 74–97. doi:10.1016/j.brainresrev.2004.08.005 15708629

[B18] Choi S.-W. Kim H. S. Kim G.-Y. Jeon Y. Park S. M. Lee J.-Y. Jung H. Y. Sohn B. K. Choi J. S. Kim D.-J. (2014). Similarities and differences among Internet gaming disorder, gambling disorder and alcohol use disorder: A focus on impulsivity and compulsivity. Journal of Behavioral Addictions, 3(4), 246–2533. doi:10.1556/JBA.3.2014.4.6 25592310 PMC4291830

[B19] Cohen J. (1992). A power primer. Psychological Bulletin, 112(1), 155–159. doi:10.1037/0033-2909.112.1.155 19565683

[B20] Cone-Wesson B. (2005). Prenatal alcohol and cocaine exposure: Influences on cognition, speech, language, and hearing. Journal of Communication Disorders, 38(4), 279–302. doi:10.1016/j.jcomdis.2005.02.004 15862811

[B21] Crews F. T. Boettiger C. A. (2009). Impulsivity, frontal lobes and risk for addiction. Pharmacology Biochemistry and Behavior, 93(3), 237–247. doi:10.1016/j.pbb.2009.04.018 PMC2730661 19410598

[B22] Dawe S. Loxton N. J. (2004). The role of impulsivity in the development of substance use and eating disorders. Neuroscience and Biobehavioral Reviews, 28(3), 343–351. doi:10.1016/j.neubiorev.2004.03.007 15225976

[B23] de Wit H. (2009). Impulsivity as a determinant and consequence of drug use: A review of underlying processes. Addiction Biology, 14(1), 22–31. doi:10.1111/j.1369-1600.2008.00129.x 18855805 PMC3640851

[B24] Demetrovics Z. Szeredi B. Rózsa S. (2008). The three-factor model of Internet addiction: The development of the Problematic Internet Use Questionnaire. Behavior Research Methods, 40(2), 563–574. doi:10.3758/BRM.40.2.563 18522068

[B25] Ding W. Sun J. Sun Y. Chen X. Zhou Y. Zhuang Z. Li L. Zhang Y. Xu J. R. Du Y. (2014). Trait impulsivity and impaired prefrontal impulse inhibition function in adolescents with Internet gaming addiction revealed by a Go/No-Go fMRI study. Behavioral and Brain Functions, 10(1), 20. doi:10.1186/1744-9081-10-20 24885073 PMC4050412

[B26] Dong G. Huang J. Du X. (2012). Alterations in regional homogeneity of resting-state brain activity in Internet gaming addicts. Behavioral and Brain Functions, 8(1), 41. doi:10.1186/1744-9081-8-41 22901705 PMC3506436

[B27] Doran N. Spring B. McChargue D. Pergadia M. Richmond M. (2004). Impulsivity and smoking relapse. Nicotine & Tobacco Research, 6(4), 641–647. doi:10.1080/14622200410001727939 15370160

[B28] Du X. Qi X. Yang Y. Du G. Gao P. Zhang Y. Qin W. Li X. Zhang Q. (2016). Altered structural correlates of impulsivity in adolescents with Internet gaming disorder. Frontiers in Human Neuroscience, 10, 4. doi:10.3389/fnhum.2016.00004 26858620 PMC4729938

[B29] Durkee T. Carli V. Floderus B. Wasserman C. Sarchiapone M. Apter A. Balazs J. A. Bobes J. Brunner R. Corcoran P. Cosman D. Haring C. Hoven C. W. Kaess M. Kahn J. P. Nemes B. Postuvan V. Saiz P. A. Värnik P. Wasserman D. (2016). Pathological Internet use and risk-behaviors among European adolescents. International Journal of Environmental Research and Public Health, 13(3), 294. doi:10.3390/ijerph13030294 PMC4808957 27005644

[B30] Ellis P. D. (2010). The essential guide to effect sizes : Statistical power, meta-analysis, and the interpretation of research results. Cambridge, NY: Cambridge University Press.

[B31] Fagerstrom K.-O. Schneider N. G. (1989). Measuring nicotine dependence: A review of the Fagerstrom Tolerance Questionnaire. Journal of Behavioral Medicine, 12(2), 159–182. doi:10.1007/BF00846549 2668531

[B32] Feng Q. Chen X. Sun J. Zhou Y. Sun Y. Ding W. Zhang Y. Zhuang Z. Xu J. Du Y. (2013). Voxel-level comparison of arterial spin-labeled perfusion magnetic resonance imaging in adolescents with Internet gaming addiction. Behavioral and Brain Functions, 9(1), 33. doi:10.1186/1744-9081-9-33 23937918 PMC3751515

[B33] Fischl B. Salat D. H. Van Der Kouwe A. J. W. Makris N. Ségonne F. Quinn B. T. Dale A. M. (2004). Sequence-independent segmentation of magnetic resonance images. NeuroImage, 23, S69–S84. doi:10.1016/j.neuroimage.2004.07.016 15501102

[B34] Fradkin Y. Khadka S. Bessette K. L. Stevens M. C. (2017). The relationship of impulsivity and cortical thickness in depressed and non-depressed adolescents. Brain Imaging and Behavior, 11(5), 1515–1525. doi:10.1007/s11682-016-9612-8 27738995

[B35] Gallinat J. Meisenzahl E. Jacobsen L. K. Kalus P. Bierbrauer J. Kienast T. Witthaus H. Leopold K. Seifert F. Schubert F. Staedtgen M. (2006). Smoking and structural brain deficits: A volumetric MR investigation. European Journal of Neuroscience, 24(6), 1744–1750. doi:10.1111/j.1460-9568.2006.05050.x 17004938

[B36] Glickman M. E. Rao S. R. Schultz M. R. (2014). False discovery rate control is a recommended alternative to Bonferroni-type adjustments in health studies. Journal of Clinical Epidemiology, 67(8), 850–857. doi:10.1016/j.jclinepi.2014.03.012 24831050

[B37] Grant J. E. Potenza M. N. Weinstein A. Gorelick D. A. (2010). Introduction to behavioral addictions. The American Journal of Drug and Alcohol Abuse, 36(5), 233–241. doi:10.3109/00952990.2010.491884 20560821 PMC3164585

[B38] Hahn C. Kim D.-J. (2014). Is there a shared neurobiology between aggression and Internet addiction disorder? Journal of Behavioral Medicine, 3(1), 12–20. doi:10.1556/JBA.3.2014.1.2 PMC4117279 25215210

[B39] Islam M. A. Hossin M. Z. (2016). Prevalence and risk factors of problematic Internet use and the associated psychological distress among graduate students of Bangladesh. Asian Journal of Gambling Issues and Public Health, 6(1), 11. doi:10.1186/s40405-016-0020-1 27942430 PMC5122610

[B40] Jaworska N. Cox S. M. Casey K. F. Boileau I. Cherkasova M. Larcher K. Dagher A. Benkelfat C. Leyton M. (2017). Is there a relation between novelty seeking, striatal dopamine release and frontal cortical thickness? PLOS One, 12(3), e0174219. doi:10.1371/journal.pone.0174219 28346539 PMC5367687

[B41] Kaag A. M. Crunelle C. L. van Wingen G. Homberg J. van den Brink W. Reneman L. (2014). Relationship between trait impulsivity and cortical volume, thickness and surface area in male cocaine users and non-drug using controls. Drug and Alcohol Dependence, 144, 210–217. doi:10.1016/j.drugalcdep.2014.09.016 25278147

[B42] Karama S. Ducharme S. Corley J. Chouinard-Decorte F. Starr J. M. Wardlaw J. M. Bastin M. E. Deary I. J. (2015). Cigarette smoking and thinning of the brain’s cortex. Molecular Psychiatry, 20(6), 778–785. doi:10.1038/mp.2014.187 25666755 PMC4430302

[B43] Klein A. Tourville J. (2012). 101 labeled brain images and a consistent human cortical labeling protocol. Frontiers in Neuroscience, 6, 171. doi:10.3389/fnins.2012.00171 23227001 PMC3514540

[B44] Ko C. H. Liu G. C. Hsiao S. Yen J. Y. Yang M. J. Lin W. C. Yen C. F. Chen C. S. (2009). Brain activities associated with gaming urge of online gaming addiction. Journal of Psychiatric Research, 43(7), 739–747. doi:10.1016/j.jpsychires.2008.09.012 18996542

[B45] Kreek M. J. Nielsen D. A. Butelman E. R. LaForge K. S. (2005). Genetic influences on impulsivity, risk taking, stress responsivity and vulnerability to drug abuse and addiction. Nature Neuroscience, 8(11), 1450–1457. doi:10.1038/nn1583 16251987

[B46] Kühn S. Gallinat J. (2015). Brains online: Structural and functional correlates of habitual Internet use. Addiction Biology, 20(2), 415–422. doi:10.1111/adb.12128 24612094

[B47] Kuss D. J. Lopez-Fernandez O. (2016). Internet addiction and problematic Internet use: A systematic review of clinical research. World Journal of Psychiatry, 6(1), 143–176. doi:10.5498/wjp.v6.i1.143 27014605 PMC4804263

[B48] Lee D. Park J. Namkoong K. Kim I. Y. Jung Y.-C. (2018). Gray matter differences in the anterior cingulate and orbitofrontal cortex of young adults with Internet gaming disorder: Surface-based morphometry. Journal of Behavioral Medicine, 7(1), 21–30. doi:10.1556/2006.7.2018.20 PMC6035012 29529887

[B49] Lee H. W. Choi J.-S. Shin Y.-C. Lee J.-Y. Jung H. Y. Kwon J. S. (2012). Impulsivity in Internet addiction: A comparison with pathological gambling. Cyberpsychology, Behavior, and Social Networking, 15(7), 373–377. doi:10.1089/cyber.2012.0063 22663306

[B50] Limbrick-Oldfield E. H. Van Holst R. J. Clark L. (2013). Review article Fronto-striatal dysregulation in drug addiction and pathological gambling: Consistent inconsistencies? NeuroImage: Clinical, 2(1), 385–393. doi:10.1016/j.nicl.2013.02.005 24179792 PMC3777686

[B51] Mak K.-K. Lai C.-M. Watanabe H. Kim D.-I. Bahar N. Ramos M. Ramos M. Young K. S. Ho R. C. Aum N. R. Cheng C. (2014). Epidemiology of Internet behaviors and addiction among adolescents in six Asian countries. Cyberpsychology, Behavior, and Social Networking, 17(11), 720–728. doi:10.1089/cyber.2014.0139 25405785

[B52] Matsuo K. Nicoletti M. Nemoto K. Hatch J. P. Peluso M. A. M. Nery F. G. Soares J. C. (2009). A voxel-based morphometry study of frontal gray matter correlates of impulsivity. Human Brain Mapping, 30(4), 1188–1195. doi:10.1002/hbm.20588 18465751 PMC6870717

[B53] Moccia L. Pettorruso M. De Crescenzo F. De Risio L. di Nuzzo L. Martinotti G. Bifone A. Janiri L. Di Nicola M. (2017). Neural correlates of cognitive control in gambling disorder: A systematic review of fMRI studies. Neuroscience and Biobehavioral Reviews, 78, 104–116. doi:10.1016/j.neubiorev.2017.04.025 28456569

[B54] Muhlert N. Lawrence A. D. (2015). Brain structure correlates of emotion-based rash impulsivity. NeuroImage, 115, 138–146. doi:10.1016/j.neuroimage.2015.04.061 25957991 PMC4463859

[B55] Oldfield R. C. (1971). The assessment and analysis of handedness: The Edinburgh Inventory. Neuropsychologia, 9(1), 97–113. doi:10.1016/0028-3932(71)90067-4 5146491

[B56] Pardoe H. R. Pell G. S. Abbott D. F. Jackson G. D. (2009). Hippocampal volume assessment in temporal lobe epilepsy: How good is automated segmentation? Epilepsia, 50(12), 2586–2592. doi:10.1111/j.1528-1167.2009.02243.x 19682030 PMC3053147

[B57] Park B. Han D. H. Roh S. (2017). Neurobiological findings related to Internet use disorders. Psychiatry and Clinical Neurosciences, 71(7), 467–478. doi:10.1111/pcn.12422 27450920

[B58] Park S. Hong K.-E. M. Park E. J. Ha K. S. Yoo H. J. (2013). The association between problematic Internet use and depression, suicidal ideation and bipolar disorder symptoms in Korean adolescents. Australian & New Zealand Journal of Psychiatry, 47(2), 153–159. doi:10.1177/0004867412463613 23047959

[B59] Patton J. H. Stanford M. S. Barratt E. S. (1995). Factor structure of the Barratt Impulsiveness Scale. Journal of Clinical Psychology, 51(6), 768–774. doi:10.1002/1097-4679(199511)51:6<768::AID-JCLP2270510607>3.0.CO;2-1 8778124

[B60] Perlaki G. Orsi G. Plozer E. Altbacker A. Darnai G. Nagy S. A. Horvath R. Toth A. Doczi T. Kovacs N. Bogner P. Schwarcz A. Janszky J. (2014). Are there any gender differences in the hippocampus volume after head-size correction? A volumetric and voxel-based morphometric study. Neuroscience Letters, 570, 119–123. doi:10.1016/j.neulet.2014.04.013 24746928

[B61] Prokhorov A. V. Pallonen U. E. Fava J. L. Ding L. Niaura R. (1996). Measuring nicotine dependence among high-risk adolescent smokers. Addictive Behaviors, 21(1), 117–127. doi:10.1016/0306-4603(96)00048-2 8729713

[B62] Robbins T. W. Ersche K. D. Everitt B. J. (2008). Drug addiction and the memory systems of the brain. Annals of the New York Academy of Sciences, 1141(1), 1–21. doi:10.1196/annals.1441.020 18991949

[B63] Schilling C. Kühn S. Romanowski A. Schubert F. Kathmann N. Gallinat J. (2012). Cortical thickness correlates with impulsiveness in healthy adults. NeuroImage, 59(1), 824–830. doi:10.1016/j.neuroimage.2011.07.058 21827861

[B64] Schulte E. M. Grilo C. M. Gearhardt A. N. (2016). Shared and unique mechanisms underlying binge eating disorder and addictive disorders. Clinical Psychology Review, 44, 125–139. doi:10.1016/j.cpr.2016.02.001 26879210 PMC5796407

[B65] Shapira N. A. Lessig M. C. Goldsmith T. D. Szabo S. T. Lazoritz M. Gold M. S. Stein D. J. (2003). Problematic Internet use: Proposed classification and diagnostic criteria. Depression and Anxiety, 17(4), 207–216. doi:10.1002/da.10094 12820176

[B66] Söderström P. Horne M. Mannfolk P. van Westen D. Roll M. (2017). Tone-grammar association within words: Concurrent ERP and fMRI show rapid neural pre-activation and involvement of left inferior frontal gyrus in pseudoword processing. Brain and Language, 174, 119–126. doi:10.1016/j.bandl.2017.08.004 28850882

[B67] Spielberger C. D. (2010). State-Trait Anxiety Inventory. In Weiner I. B. Craighead W. E. (Eds.), The Corsini encyclopedia of psychology (4th ed.). Hoboken, NJ: John Wiley & Sons, Inc.

[B68] Stevens L. Verdejo-García A. Goudriaan A. E. Roeyers H. Dom G. Vanderplasschen W. (2014). Impulsivity as a vulnerability factor for poor addiction treatment outcomes: A review of neurocognitive findings among individuals with substance use disorders. Journal of Substance Abuse Treatment, 47(1), 58–72. doi:10.1016/j.jsat.2014.01.008 24629886

[B69] Sun D.-L. Chen Z.-J. Ma N. Zhang X.-C. Fu X.-M. Zhang D.-R. (2009). Decision-making and prepotent response inhibition functions in excessive Internet users. CNS Spectrums, 14(2), 75–81. doi:10.1017/S1092852900000225 19238122

[B70] Swick D. Ashley V. Turken A. U. (2008). Left inferior frontal gyrus is critical for response inhibition. BMC Neuroscience, 9(1), 102. doi:10.1186/1471-2202-9-102 18939997 PMC2588614

[B71] Tényi D. Gyimesi C. Kovács N. Tényi T. Janszky J. (2016). The possible role of the insula in the epilepsy and the gambling disorder of Fyodor Dostoyevsky. Journal of Behavioral Addictions, 5(3), 542–547. doi:10.1556/2006.5.2016.061 27558486 PMC5264423

[B72] Tu P. C. Kuan Y. H. Li C. T. Su T. P. (2017). Structural correlates of trait impulsivity in patients with bipolar disorder and healthy controls: A surface-based morphometry study. Psychological Medicine, 47(7), 1292–1299. doi:10.1017/S0033291716003299 28077175

[B73] Van Rooij A. J. Prause N. (2014). A critical review of “Internet addiction” criteria with suggestions for the future. Journal of Behavioral Addictions, 3(4), 203–213. doi:10.1556/JBA.3.2014.4.1 25592305 PMC4291825

[B74] Verdejo-García A. Bechara A. (2009). A somatic marker theory of addiction. Neuropharmacology, 56(Suppl. 1), 48–62. doi:10.1016/j.neuropharm.2008.07.035 18722390 PMC2635337

[B75] Verhoeven K. J. F. Simonsen K. L. McIntyre L. M. (2005). Implementing false discovery rate control: Increasing your power. Oikos, 108(3), 643–647. doi:10.1111/j.0030-1299.2005.13727.x

[B76] Weinstein A. Lejoyeux M. (2010). Internet addiction or excessive Internet use. The American Journal of Drug and Alcohol Abuse, 36(5), 277–283. doi:10.3109/00952990.2010.491880 20545603

[B77] Wu A. M. S. Cheung V. I. Ku L. Hung E. P. W. (2013). Psychological risk factors of addiction to social networking sites among Chinese smartphone users. Journal of Behavioral Addictions, 2(3), 160–166. doi:10.1556/JBA.2.2013.006 PMC4117295 25215198

[B78] Xue G. Lu Z. Levin I. P. Bechara A. (2010). The impact of prior risk experiences on subsequent risky decision-making: The role of the insula. NeuroImage, 50(2), 709–716. doi:10.1016/j.neuroimage.2009.12.097 20045470 PMC2828040

[B79] Yau Y. H. C. Potenza M. N. White M. A. (2012). Problematic Internet use, mental health and impulse control in an online survey of adults. Journal of Behavioral Addictions, 2(2), 72–81. doi:10.1556/JBA.1.2012.015 PMC3840434 24294501

[B80] Yuan K. Cheng P. Dong T. Bi Y. Xing L. Yu D. Zhao L. Dong M. von Deneen K. M. Liu Y. Qin W. Tian J. (2013). Cortical thickness abnormalities in late adolescence with online gaming addiction. PLoS One, 8(1), e53055. doi:10.1371/journal.pone.0053055 23326379 PMC3541375

